# High Current CD4+ T Cell Count Predicts Suboptimal Adherence to Antiretroviral Therapy

**DOI:** 10.1371/journal.pone.0140791

**Published:** 2015-10-15

**Authors:** Alexander O. Pasternak, Marijn de Bruin, Margreet Bakker, Ben Berkhout, Jan M. Prins

**Affiliations:** 1 Laboratory of Experimental Virology, Department of Medical Microbiology, Center for Infection and Immunity Amsterdam (CINIMA), Academic Medical Center of the University of Amsterdam, Amsterdam, The Netherlands; 2 Health Psychology Group, Institute of Applied Health Sciences, University of Aberdeen, Aberdeen, United Kingdom; 3 Department of Internal Medicine, Division of Infectious Diseases, Academic Medical Center of the University of Amsterdam, Amsterdam, The Netherlands; Infectious Disease Service, UNITED STATES

## Abstract

High levels of adherence to antiretroviral therapy (ART) are necessary for achieving and maintaining optimal virological suppression, as suboptimal adherence leads to therapy failure and disease progression. It is well known that adherence to ART predicts therapy response, but it is unclear whether clinical outcomes of ART predict adherence. To examine the predictive power of current CD4^+^ T cell count for adherence of HIV-infected individuals to ART, we performed a cross-sectional analysis of 133 Dutch HIV patients with electronically measured adherence. In a multivariate analysis adjusting for a number of sociodemographic and clinical variables, high current CD4^+^ T cell count (>660 cells/mm^3^) was most strongly associated with lower adherence to ART (assessed as a continuous variable) during a two-month period immediately following the measurements of variables (*P* = 0.008). The twice-per-day (versus once-per-day) dosing regimen was also significantly associated with lower adherence (*P* = 0.014). In a second multivariate analysis aimed at determining the predictors of suboptimal (<100% of the doses taken) adherence, high current CD4^+^ T cell count was again the strongest independent predictor of suboptimal adherence to ART (*P* = 0.015), and the twice-per-day dosing regimen remained associated with suboptimal adherence (*P* = 0.025). The association between suboptimal adherence and virological suppression was significant in patients with high CD4^+^ T cell counts, but not in patients with low or intermediate CD4^+^ T cell counts (*P* = 0.036 and *P* = 0.52, respectively; *P* = 0.047 for comparison of the effects of adherence on virological suppression between patients with high vs. low or intermediate CD4^+^ T cell counts), suggesting that apart from promoting suboptimal adherence, high CD4^+^ T cell count also strengthens the effect of adherence on virological suppression. Therefore, sustained efforts to emphasize continued adherence are necessary, especially for patients with high CD4^+^ T cell counts.

## Introduction

High levels of adherence to antiretroviral therapy (ART) are necessary for achieving and maintaining optimal virological suppression [1,2], as suboptimal adherence leads to therapy failure and disease progression [3,4]. It is well known that adherence to ART predicts therapy response [5,6]. However, it is unclear whether the opposite is true, i.e. whether clinical outcomes of ART (plasma viral load and CD4^+^ T cell count) predict adherence.

The CD4^+^ T cell count is an important measure of clinical outcome of therapy, and a good immunological response is an integral part of success on ART. High CD4^+^ count, coupled to undetectable plasma viral load, delivers a reassuring message of success on ART to the patient, whereas low CD4^+^ count despite virological suppression may signal that the therapy is not working as it should. Because the patients’ perceptions of ART influence adherence [7,8], CD4^+^ count may therefore be a predictive factor for adherence to therapy. However, it is difficult to speculate about the exact effect that CD4^+^ count could have on adherence. For example, a high CD4^+^ count, signaling therapy success, may either encourage a patient to be more adherent, or it may, on the contrary, result in the decreased attention to taking all antiretroviral drugs each day. In the latter case, timely behavioral intervention is important.

Surprisingly, to date no study has specifically addressed, and very few studies have mentioned, the influence of current CD4^+^ count on adherence. Moreover, studies that did report such an association provided conflicting results [9,10]. The aim of the current study was to examine the relationship between current CD4^+^ count and electronically monitored adherence to ART, while controlling for a number of sociodemographic and clinical variables, in 133 Dutch patients on suppressive ART.

## Methods

We conducted a cross-sectional analysis of 133 Dutch patients visiting the HIV outpatient clinic of the Academic Medical Center (Amsterdam, the Netherlands) in 2005–2006, who were participating in a randomized controlled trial investigating the effects of a behavioral intervention to increase adherence. The results of the trial were reported previously [11]. Patients were eligible if they were older than 18 years, treatment-experienced (>6 months on ART), started antiretroviral treatment in 1996 or later, and had no uncontrolled psychiatric or drug addiction problems that would prevent them from being able to comply with the study procedures (patients with such problems constitute an extremely small part of our patient population: out of 348 screened patients, 5 and 7 patients were not approached because of severe psychological and drug addiction problems, respectively). Since the nurses preferred to apply this new intervention initially without the additional challenge of language and/or cultural barriers, the trial was focused on native Dutch patients (the main patient population in the Netherlands). The study was approved by the Medical Ethical Committee of the Academic Medical Center, and all patients provided written informed consent. The study has been conducted in accordance with the ethical principles in the Declaration of Helsinki.

The time period analyzed in the current study corresponds to a two-month adherence monitoring period between patient inclusion and randomization [11]. Besides measuring adherence, no interventions took place during this period. The subsequent group allocation and adherence interventions could not influence the results of the present study and are not reported here. The following sociodemographic and clinical variables were collected at inclusion: age, gender, education level, current employment, ART regimen, dosing regimen, time on ART, time on ART with undetectable plasma viral load, current CD4^+^ T cell count, nadir CD4^+^ T cell count, and current plasma viral load detectability. Adherence was measured electronically using MEMS-cap pill bottles (Aardex, Switzerland) during two-month periods immediately following the assessment of variables. MEMS-cap pill bottles recorded the moments of bottle opening electronically. Adherence as measured by MEMS was defined as the percentage of prescribed doses actually taken during the assessment period, and was calculated by dividing the number of times the pill bottle had been opened by the number of times it should have been opened, multiplied by 100.

Explanatory power of the sociodemographic and clinical variables was examined both for adherence as a continuous variable and for <100% adherence versus 100% adherence. This cutoff was based on fitting receiver operating characteristic (ROC) curve and Fisher’s exact tests to determine the most clinically significant cutoff value of adherence for virological suppression. Continuous predictors were dichotomized by tertiles to high, intermediate, and low categories with intermediate category used as a reference category. For the univariate analysis of adherence as a continuous variable, nonparametric Mann-Whitney tests were used to compare adherence values between different categories of a given predictor, and a generalized linear model (GeLM) using a rank-transformed dependent variable was fitted for the multivariate analysis. To analyze the predictive value of variables for suboptimal adherence (<100%), we used logistic regression (enter method). Fisher’s exact tests were used to determine effects of CD4^+^ count on virological suppression and effects of adherence on virological suppression in different CD4^+^ count strata. Cochran–Mantel–Haenszel test was used to compare the effects of adherence on virological suppression between patients with high vs. low or intermediate CD4^+^ T cell counts. GeLM, ROC curve analysis, and logistic regression were performed using IBM SPSS Statistics 22 (available at: http://www.spss.com). Mann-Whitney and Fisher’s tests were performed using GraphPad Prism 5.01 (available at: http://www.graphpad.com). Cochran–Mantel–Haenszel test was performed using a spreadsheet available at http://www.biostathandbook.com/cmh.html. All statistical tests were two-sided. *P* values of <0.05 were considered statistically significant.

## Results

Patient characteristics are shown in Table 1. Median age was 47.2 years and 91% were males. At the moment of measurements, patients had been treated with suppressive ART for a median of 50.6 months (median 41.6 months of continuous plasma viral load suppression) and had a median CD4^+^ T cell count of 590 cells/mm^3^. Median CD4^+^ T cell count nadir was 150 cells/mm^3^. Low-level plasma viral load was detectable (>50 copies/ml) in 7.1% of patients. Adherence was high in this patient cohort: 73.7% of patients demonstrated adherence of >95% and 51.1% of patients were 100% adherent. Only 9.0% of patients demonstrated <80% adherence.

**Table 1:** Characteristics of the patients.

Age, years (n = 133)		47.2 (40.9–54.6)^1^
Gender (n = 133)	Female	9.0 (12/133)
	Male	91.0 (121/133)
Transmission route (n = 120)	Heterosexual	19.2 (23/120)
	Intravenous drug use	0.8 (1/120)
	MSM	80.0 (96/120)
Education (n = 128)	Low	25.8 (33/128)
	Middle	35.9 (46/128)
	High	38.3 (49/128)
Currently employed (n = 122)	Yes	68.0 (83/122)
	No	32.0 (39/122)
Antiretroviral therapy (n = 128)	NNRTI-based	60.2 (77/128)
	PI-based	28.9 (37/128)
	Other^2^	10.9 (14/128)
Dosing regimen (n = 133)	One dose/day	60.2 (80/133)
	Two doses/day	39.8 (53/133)
Time on therapy, months (n = 129)		50.6 (19.5–90.5)
Time on therapy with undetectable plasma viral load, months (n = 129)		41.6 (15.8–76.2)
Current CD4^+^ T cell count, cells/mm^3^ (n = 127)		590 (380–720)
CD4^+^ T cell count nadir, cells/mm^3^ (n = 128)		150 (60–227)
Current plasma viral load (n = 127)	>50 copies/ml	7.1 (9/127)
	<50 copies/ml	92.9 (118/127)
Adherence to ART, % (n = 133)		100 (94.7–100)
	<80%	9.0 (12/133)
	80–94%	17.3 (23/133)
	95–99%	22.6 (30/133)
	100%	51.1 (68/133)

^1^Data are medians (interquartile ranges) for continuous variables and % (proportions) for discrete variables.

^2^Triple NRTI (n = 9), PI+NNRTI-based (n = 5).

We started with analyzing the factors associated with adherence as a continuous variable. In the univariate analysis, a twice-per-day dosing regimen, heterosexual transmission route, and high current CD4^+^ T cell count (highest tertile: >660 cells/mm^3^) were significantly associated with lower adherence (Table 2). In the multivariate analysis, high current CD4^+^ T cell count and twice-per-day dosing regimen remained associated with lower adherence (*P* = 0.008 and *P* = 0.014, respectively). High CD4^+^ T cell count nadir and longer time on therapy with undetectable plasma viral load were associated with higher adherence with borderline significance (*P* = 0.034 and *P* = 0.045, respectively).

**Table 2:** Factors associated with adherence to ART (measured as continuous variable).

Variable		Adherence, %^1^	*P*	Adjusted *P*
Age (n = 133)	<42 years (n = 46)	100 (94.2–100)	0.49	0.27
	42–52 years (n = 42)	98.2 (94.4–100)		
	>52 years (n = 45)	100 (95.6–100)	0.27	0.60
Gender (n = 133)	Female (n = 12)	96.4 (88.9–100)	0.14	0.69
	Male (n = 121)	100 (95.5–100)		
Transmission route	Heterosexual (n = 23)	96.5 (88.4–100)		
(n = 120)	Intravenous drug use (n = 1)	96.9		
	MSM (n = 96)	100 (96.4–100)	0.033	0.67
Education (n = 128)	Low (n = 33)	98.2 (94.2–100)	0.79	0.30
	Middle (n = 46)	98.7 (91.3–100)		
	High (n = 49)	100 (96.9–100)	0.12	0.73
Currently employed	Yes (n = 83)	100 (95.5–100)		
(n = 122)	No (n = 39)	100 (96.5–100)	0.44	0.73
Antiretroviral therapy	NNRTI (n = 77)	99.2 (94.7–100)		
(n = 128)	PI (n = 37)	100 (96.7–100)	0.39	0.44
	Other (n = 14)	98.2 (93.3–100)	0.88	0.96
Dosing regimen	One dose/day (n = 80)	100 (96.4–100)		
(n = 133)	Two doses/day (n = 53)	98.2 (92.4–100)	0.036	0.014
Time on	<30 months (n = 43)	99.2 (95.5–100)	0.37	0.93
therapy (n = 129)	30–72 months (n = 44)	98.2 (91.9–100)		
	>72 months (n = 42)	100 (95.7–100)	0.26	0.40
Time on therapy with	<24 months (n = 41)	99.2 (95.1–100)	0.49	0.84
undetectable plasma	24–65 months (n = 46)	98.2 (92.6–100)		
viral load (n = 129)	>65 months (n = 42)	100 (96.0–100)	0.25	0.045
Current CD4^+^ T cell	<450 cells/mm^3^ (n = 43)	100 (97.3–100)	0.71	0.65
count (n = 127)	450–660 cells/mm^3^ (n = 39)	100 (96.5–100)		
	>660 cells/mm^3^ (n = 45)	97.3 (91.5–100)	0.030	0.008
CD4^+^ T cell count	<100 cells/mm^3^ (n = 41)	100 (96.5–100)	0.26	0.29
nadir (n = 128)	100–200 cells/mm^3^ (n = 46)	98.7 (90.6–100)		
	>200 cells/mm^3^ (n = 41)	100 (96.4–100)	0.24	0.034
Current plasma viral	>50 copies/ml (n = 9)	96.5 (73.2–100)	0.19	0.35
load (n = 127)	<50 copies/ml (n = 118)	100 (94.7–100)		

^1^Data are medians (interquartile ranges).

Because we sought to determine the predictors of a clinically significant adherence level, we next established the most clinically significant cutoff value of adherence for prediction of detectable plasma viral load. To this end, we used the plasma viral load values measured directly after the adherence assessment period. The proportion of virologically suppressed patients (plasma viral load <50 copies/ml) was 83.6% in all patients, 66.7% in patients with <80% adherence, 78.3% in patients with 80–94% adherence, 76.7% in patients with 95–99% adherence, and 92.1% in patients with 100% adherence (S1 Fig). This suggested that 100% might be the optimal adherence level in this cohort. Indeed, the ROC curve analysis revealed that 100% was the most clinically significant cutoff value of adherence for prediction of detectable plasma viral load (S1 Table). This was confirmed by direct comparison of different adherence cutoff values (Table 3): 100% was the only cutoff value that could significantly predict detectable plasma viral load (OR (95% CI) = 3.79 (1.29–11.1), *P* = 0.016).

**Table 3:** Comparison of different adherence cutoff values for the prediction of detectable plasma viral load.

Adherence threshold	All patients (n = 128)	Patients with pVL^1^>50 copies/ml (n = 21)	Patients with pVL<50 copies/ml (n = 107)	OR (95% CI)	*P*
80%	90.1 (116/128)	81.0 (17/21)^2^	92.5 (99/107)	2.91 (0.79–10.8)	0.11
85%	89.1 (114/128)	81.0 (17/21)	90.7 (97/107)	2.28 (0.64–8.12)	0.25
90%	82.8 (106/128)	81.0 (17/21)	83.2 (89/107)	1.16 (0.35–3.87)	0.76
95%	72.7 (93/128)	57.1 (12/21)	75.7 (81/107)	2.34 (0.89–6.17)	0.11
100%	49.2 (63/128)	23.8 (5/21)	54.2 (58/107)	3.79 (1.29–11.1)	0.016

^1^pVL, plasma viral load.

^2^Shown are % (proportions) of patients with adherence higher than or equal to the respective adherence threshold.

Next, we determined the predictors of suboptimal (<100%) adherence to ART. In the univariate analysis, high current CD4^+^ T cell count (>660 cells/mm^3^) and a twice-per-day dosing regimen were significantly associated with suboptimal adherence (Table 4). Both these factors remained significantly predictive of suboptimal adherence in the multivariate model (OR (95% CI): 6.21 (1.42–27.0), *P* = 0.015 and 3.27 (1.16–9.17), *P* = 0.025, respectively). Therefore, high current CD4^+^ T cell count was the strongest independent predictor of suboptimal adherence.

**Table 4:** Factors associated with suboptimal adherence to ART.

Variable		All patients	Patients with 100%	Patients with <100%	OR (95% CI)	*P*	Adjusted OR	*P*
		(n = 133)	adherence (n = 68)	adherence (n = 65)			(95% CI)	
Age (n = 133)		47.2 (40.9–54.6)^1^	48.1 (40.5–54.8)	44.9 (41.2–53.8)		0.72		
	<42 years	34.6 (46/133)	36.8 (25/68)	32.3 (21/65)	0.63 (0.27–1.46)	0.28	0.44 (0.13–1.48)	0.18
	42–52 years	31.6 (42/133)	26.5 (18/68)	36.9 (24/65)	1.00		1.00	
	>52 years	33.8 (45/133)	36.8 (25/68)	30.8 (20/65)	0.60 (0.26–1.40)	0.24	0.95 (0.27–3.34)	0.93
Gender (n = 133)	Female	9.0 (12/133)	5.9 (4/68)	12.3 (8/65)	2.25 (0.64–7.87)	0.21	1.31 (0.18–9.43)	0.79
	Male	91.0 (121/133)	94.1 (64/68)	87.7 (57/65)	1.00		1.00	
Education (n = 128)	Low	25.8 (33/128)	21.2 (14/66)	30.6 (19/62)	1.24 (0.51–3.06)	0.64	1.82 (0.47–7.14)	0.39
	Middle	35.9 (46/128)	33.3 (22/66)	38.7 (24/62)	1.00		1.00	
	High	38.3 (49/128)	45.5 (30/66)	30.6 (19/62)	0.58 (0.26–1.31)	0.19	0.73 (0.24–2.21)	0.58
Currently employed	Yes	68.0 (83/122)	65.2 (43/66)	71.4 (40/56)	1.00		1.00	
(n = 122)	No	32.0 (39/122)	34.8 (23/66)	28.6 (16/56)	0.75 (0.35–1.62)	0.46	0.69 (0.22–2.20)	0.53
Antiretroviral therapy	NNRTI	60.2 (77/128)	59.4 (38/64)	60.9 (39/64)	1.00		1.00	
(n = 128)	PI	28.9 (37/128)	31.3 (20/64)	26.6 (17/64)	0.83 (0.38–1.82)	0.64	0.89 (0.29–2.77)	0.84
	other	10.9 (14/128)	9.4 (6/64)	12.5 (8/64)	1.30 (0.41–4.10)	0.66	1.56 (0.30–8.20)	0.60
Dosing regimen	One dose/day	60.2 (80/133)	70.6 (48/68)	49.2 (32/65)	1.00		1.00	
(n = 133)	Two doses/day	39.8 (53/133)	29.4 (20/68)	50.8 (33/65)	2.48 (1.21–5.05)	0.013	3.27 (1.16–9.17)	0.025
Transmission route	Heterosexual	19.2 (23/120)	13.3 (8/60)	25.0 (15/60)	1.00		1.00	
(n = 120)	IV drug use	0.8 (1/120)	0.0 (0/60)	1.7 (1/60)				
	MSM	80.0 (96/120)	86.7 (52/60)	73.3 (44/60)	0.45 (0.18–1.16)	0.10	0.87 (0.18–4.13)	0.86
Time on		50.6 (19.5–90.5)	49.1 (19.0–94.9)	54.5 (19.9–83.2)		0.82		
therapy (n = 129)	<30 months	33.3 (43/129)	32.3 (21/65)	34.4 (22/64)	0.87 (0.38–2.03)	0.75	0.73 (0.10–5.41)	0.75
	30–72 months	34.1 (44/129)	30.8 (20/65)	37.5 (24/64)	1.00		1.00	
	>72 months	32.6 (42/129)	36.9 (24/65)	28.1 (18/64)	0.63 (0.27–1.46)	0.28	1.94 (0.36–10.3)	0.44
Time on therapy with		41.6 (15.8–76.2)	40.4 (16.3–82.0)	41.7 (15.1–67.3)		0.59		
undetectable plasma	<24 months	31.8 (41/129)	30.8 (20/65)	32.8 (21/64)	0.88 (0.38–2.05)	0.77	2.21 (0.32–15.2)	0.42
viral load (n = 129)	24–65 months	35.7 (46/129)	32.3 (21/65)	39.1 (25/64)	1.00		1.00	
	>65 months	32.6 (42/129)	36.9 (24/65)	28.1 (18/64)	0.63 (0.27–1.46)	0.28	0.18 (0.03–1.23)	0.080
Current CD4^+^ T cell		590 (380–720)	560 (393–670)	610 (370–730)		0.61		
count (n = 127)	<450 cells/mm^3^	33.9 (43/127)	35.9 (23/64)	31.7 (20/63)	1.39 (0.58–3.36)	0.46	0.96 (0.25–3.62)	0.95
	450–660 cells/mm^3^	30.7 (39/127)	37.5 (24/64)	23.8 (15/63)	1.00		1.00	
	>660 cells/mm^3^	35.4 (45/127)	26.6 (17/64)	44.4 (28/63)	2.64 (1.09–6.37)	0.031	6.21 (1.42–27.0)	0.015
CD4^+^ T cell count		150 (60–227)	150 (60–250)	150 (60–210)		0.89		
nadir (n = 128)	<100 cells/mm^3^	32.0 (41/128)	32.3 (21/65)	31.7 (20/63)	0.80 (0.34–1.86)	0.60	0.88 (0.29–2.75)	0.83
	100–200 cells/mm^3^	35.9 (46/128)	32.3 (21/65)	39.7 (25/63)	1.00		1.00	
	>200 cells/mm^3^	32.0 (41/128)	35.4 (23/65)	28.6 (18/63)	0.66 (0.28–1.53)	0.33	0.36 (0.10–1.27)	0.11
Current plasma viral	>50 copies/ml	7.1 (9/127)	4.7 (3/64)	9.5 (6/63)	2.14 (0.51–8.93)	0.30	1.88 (0.30–11.9)	0.50
load (n = 127)	<50 copies/ml	92.9 (118/127)	95.3 (61/64)	90.5 (57/63)	1.00		1.00	

^1^Data are % (proportion) of patients or median value (interquartile range).

Next, we determined whether the effect of high current CD4^+^ T cell count on adherence translates into the effect of high current CD4^+^ T cell count on virological suppression. Immediately after the adherence assessment period, plasma viral load was detectable in 8 out of 43 (18.6%) patients with high CD4^+^ count, compared with 11 out of 79 (13.9%) patients with low or intermediate CD4^+^ count. Thus, the overall contribution of high current CD4^+^ T cell count to the detectability of plasma viral load was insignificant (OR (95% CI): 1.41 (0.52–3.83), *P* = 0.60). Finally, to determine whether the effects of adherence on virological suppression differed among patients with high versus low or intermediate CD4^+^ T cell counts, we stratified the patients based on their CD4^+^ counts and analyzed the predictive value of suboptimal adherence for plasma viral load detectability separately in both strata (Table 5). In patients with low or intermediate CD4^+^ counts, 17.1% and 11.4% of patients with suboptimal and optimal adherence, respectively, had detectable plasma viral load (*P* = 0.52). However, in patients with high CD4^+^ counts, 28.6% of patients with suboptimal adherence, but no patients with optimal adherence, had detectable plasma viral load (*P* = 0.036). The effect of adherence on virological suppression was stronger in patients with high vs. low or intermediate CD4^+^ counts (*P* = 0.047). Therefore, apart from promoting suboptimal adherence, high CD4^+^ T cell count also appeared to boost the effect of adherence on virological suppression.

**Table 5:** Effects of adherence on virological suppression among patients with high versus low or intermediate CD4+ T cell counts.

		Adherence <100%	Adherence 100%	*P*
CD4+ T cell count	pVL^1^ <50 copies/ml	29^2^	39	0.52
≤660 cells/mm^3^	pVL >50 copies/ml	6	5	
CD4+ T cell count	pVL <50 copies/ml	20	15	0.036
>660 cells/mm^3^	pVL >50 copies/ml	8	0	

^1^pVL, plasma viral load.

^2^Patient numbers are shown.

## Discussion

A number of factors associated with suboptimal adherence to ART have been identified previously, such as younger age, greater pill burden, lower education level, side effects of therapy, and interference with other activities [2,10,12,13,14]. Patients’ beliefs about ART and perceptions of therapy success or failure have also been shown to predict adherence [7,8]. As clinical outcomes may contribute to patients’ perceptions of therapy and its effects and thus may influence subsequent behavior [15], CD4^+^ counts could be expected to influence adherence. Indeed, in our cohort, patients with high (>660 cells/mm^3^) current CD4^+^ counts were, on average, six times more likely to demonstrate suboptimal adherence as compared to patients with intermediate (450–660 cells/mm^3^) CD4^+^ T cell counts. Interestingly, patients with intermediate CD4^+^ counts did not demonstrate higher rates of suboptimal adherence compared to patients with low (<450 cells/mm^3^) CD4^+^ T cell counts. This suggests that the relationship between CD4^+^ count and adherence is nonlinear and that only patients with relatively high CD4^+^ counts demonstrate suboptimal adherence. Further studies in larger cohorts are necessary to address these issues.

To our knowledge, very few studies have previously reported an association between current CD4^+^ count on ART and adherence to therapy. One study [9] reported higher rates of suboptimal adherence by higher current CD4^+^ count, whereas another study reported the opposite effect [10]. However, in these studies adherence was monitored by self-reporting, which is known to be imprecise and to overestimate adherence [3]. In contrast, adherence was monitored electronically in our study. Electronic monitoring was shown to be more sensitive than self-reporting for the detection of nonadherence [1]. In addition, half of the patients in the study by O’Connor et al. [10] received interrupted ART guided by the CD4^+^ count, whereas our patients had been on continuous ART for a median of 50.6 months before they entered the study and did not interrupt therapy throughout the study. It is possible that this can explain the difference between the effects of current CD4^+^ T cell count on adherence observed by O’Connor et al. and our study. Interestingly, Venkatesh et al. reported higher odds of suboptimal adherence by higher current CD4^+^ count and by longer time on ART [9]. They argued that treatment exhaustion was responsible for the observed effects. In contrast, treatment fatigue was unlikely to mediate the effect of higher current CD4^+^ count on adherence observed in our study, as longer time on ART was not associated with poor adherence. This is in line with the results of Cambiano et al. who studied long-term trends in adherence to ART in >2,000 patients and found that adherence does not decrease with time on therapy [16]. However, another group did report a decrease in adherence with time [17].

Several groups studied an effect of baseline (pre-therapy) CD4^+^ count on the subsequent adherence to ART. Some studies reported an association between higher (>200 or >250 cells/mm^3^) baseline CD4^+^ count and lower adherence [18], whereas others could not find such an effect [2,19]. A recent study found that higher baseline CD4^+^ count predicted treatment interruptions but not lower average adherence [19]. In our study, unlike high current CD4^+^ count, high nadir CD4^+^ count (which for most patients coincided with the moment of ART initiation) was not associated with suboptimal adherence. This might reflect the fact that our patients on average had been more than 4 years on therapy before the study was initiated and their behavior might therefore have been more dependent on the perception of their current than of their pre-therapy health status.

Besides the high current CD4^+^ count, the only variable significantly associated with suboptimal adherence in the multivariate analysis was twice-per-day dosing regimen, as compared with the once-daily regimen, confirming other studies that have reported decreasing adherence as the number of pills per day increases [10,20,21].

In this cohort, 100% adherence was found to be the only significant cutoff value of adherence for prediction of detectable plasma viral load. Also, levels of virological suppression in patients with 100% adherence were higher than in other adherence strata, including patients with 95–99% adherence (S1 Fig). This is in line with an earlier study by Paterson et al. that has found that patients with adherence of 95% or higher had better virological outcomes than patients with lower adherence [2]. However, some more recent studies have found that lower adherence thresholds (sometimes as low as 55–70%, depending on the ART regimen) might be more clinically significant, as rates of virological rebound in plasma were not found to be higher in patients with modest nonadherence [22,23]. The much more strict adherence threshold of 100% that was found to be clinically significant in our study could in part be explained by the high overall adherence level of our cohort, which could have resulted in a decreased statistical power to detect significant effects at lower adherence levels. However, our results are in line with a recent large study that examined relations between adherence and virological suppression in 21,865 patients [24]. That study found a steady increase in proportion of virologically suppressed patients with the increase in adherence up to 95% and higher, and a distinction was made between patients treated with nonnucleoside reverse transcriptase inhibitors (NNRTI) and protease inhibitors (PI): whereas adherence levels of >85% were forgivable for the former, the latter had to demonstrate >95% adherence to achieve optimal virological suppression. Interestingly, in our study, a similar difference in the adherence-virological suppression relationship was found between NNRTI- and PI-users (S1 Fig), although 100% adherence was still associated with the highest levels of virological suppression in both strata.

Although CD4^+^ T cell count was not significantly associated with virological suppression, it was found to strengthen the effects of suboptimal adherence on the virological suppression. These effects were significant in patients with high CD4^+^ T cell counts but not in patients with low or intermediate CD4^+^ T cell counts. It is possible that in patients with lower CD4^+^ counts, other factors, such as increased immune activation [25], influence the residual virus production/replication, masking the effects of suboptimal adherence on virological suppression, whereas in patients with high CD4^+^ counts, these factors are insignificant or absent.

Our study has some limitations. One limitation is that it was focused on native Dutch patients only. The observed effect of CD4^+^ count on adherence may be different among patients of non-European descent treated in the Netherlands. In general, the results of this study are applicable only to similar populations as described in this report. Another limitation is that the patients included in this analysis had chosen to enroll in a behavioral intervention to increase adherence, and while data for this study were collected before the intervention began, it is still important to consider that patients may have made changes in their adherence to therapy after inclusion. Another limitation relates to our adherence measurement method: as MEMS only indicates if the bottle was open and not if the medication was actually taken, we cannot exclude some slight overestimation of adherence due to “curiosity events”. A final limitation is that our study was not designed to detect a behavioral mechanism that is mediating the effect of high CD4^+^ count on adherence. Thus, our results warrant further studies to establish such underlying mechanisms.

In conclusion, in this cohort of 133 patients with relatively long time on ART, good immune reconstitution, good virological suppression, and high overall electronically measured adherence, high current CD4^+^ T cell count and twice-per-day dosing regimen significantly predicted suboptimal adherence. It is possible that patients who are aware of their high CD4^+^ count (interpreted as clinical success) may pay less attention to taking medications than patients with lower CD4^+^ counts, as they feel that it is less crucial to maintain optimal adherence. However, as we and others have shown that even modest nonadherence to ART may result in residual virus replication [26,27], sustained efforts to emphasize continued adherence over time are necessary, especially for patients with high CD4^+^ T cell counts.

## Supporting Information

S1 Fig Proportion of patients with virological suppression per adherence category. (PDF)

S1 Table ROC curve analysis for determination of the most clinically significant cutoff value of adherence for prediction of detectable plasma viral load. (PDF)
