# Supplementary figures and images for: High Current CD4+ T Cell Count Predicts Suboptimal Adherence to Antiretroviral Therapy
Source: PLoS One. 2015 Oct 15;10(10):e0140791. doi: 10.1371/journal.pone.0140791 (PMC4607457; doi:10.1371/journal.pone.0140791)

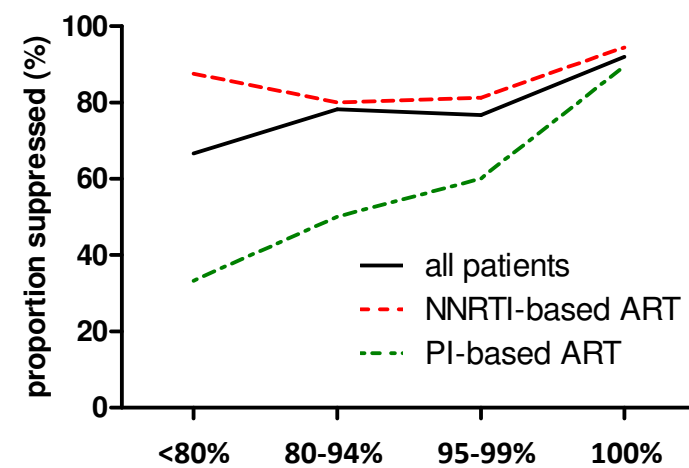

**S1 Fig.** Proportion of patients with virological suppression per adherence category.

Supplement: S1 Fig — (PDF) [file pone.0140791.s001.pdf]
