# Supplementary material for: High Current CD4+ T Cell Count Predicts Suboptimal Adherence to Antiretroviral Therapy
Source: PLoS One. 2015 Oct 15;10(10):e0140791. doi: 10.1371/journal.pone.0140791 (PMC4607457; doi:10.1371/journal.pone.0140791)
Supplement: S1 Table — (PDF) [file pone.0140791.s002.pdf]

S1 Table. ROC curve analysis for determination of the most clinically significant cutoff value of adherence for prediction of detectable plasma viral load.

Adherence cutoff, %	Sensitivity	1 - Specificity	Youden's J statistic	Distance to (0, 1)
62,4	1	1	0	1,000
63,85	1	0,962	0,038	0,962
67,05	0,991	0,962	0,029	0,962
71,5	0,991	0,923	0,068	0,923
73,825	0,981	0,923	0,058	0,923
74,725	0,972	0,923	0,049	0,923
75,25	0,963	0,923	0,04	0,924
76,125	0,953	0,923	0,03	0,924
76,775	0,953	0,885	0,068	0,886
77,7	0,944	0,885	0,059	0,887
79,025	0,935	0,885	0,05	0,887
79,475	0,925	0,885	0,04	0,888
81,7	0,925	0,846	0,079	0,849
83,925	0,916	0,846	0,07	0,850
84,7	0,907	0,846	0,061	0,851
85,575	0,897	0,846	0,051	0,852
85,725	0,879	0,846	0,033	0,855
86,175	0,869	0,846	0,023	0,856
87,5	0,86	0,846	0,014	0,858
88,8375	0,85	0,846	0,004	0,859
89,2875	0,841	0,846	-0,005	0,861
89,75	0,832	0,846	-0,014	0,863
90,6	0,822	0,846	-0,024	0,865
91,05	0,813	0,846	-0,033	0,866
91,55	0,804	0,846	-0,042	0,868
92,425	0,794	0,846	-0,052	0,871
93,3	0,794	0,808	-0,014	0,834
94,2	0,794	0,769	0,025	0,796
95,075	0,757	0,654	0,103	0,698
95,95	0,748	0,615	0,133	0,665
96,425	0,729	0,615	0,114	0,672
96,675	0,692	0,615	0,077	0,688
97,1	0,682	0,615	0,067	0,692
97,7	0,664	0,615	0,049	0,701
98,15	0,664	0,577	0,087	0,668
98,65	0,561	0,462	0,099	0,637
99,15	0,542	0,423	0,119	0,623
**100,0**	**0,542**	**0,385**	**0,157**	**0,598**
